# Juvenile Spider Mites Induce Salicylate Defenses, but Not Jasmonate Defenses, Unlike Adults

**DOI:** 10.3389/fpls.2020.00980

**Published:** 2020-07-10

**Authors:** Jie Liu, Saioa Legarrea, Juan M. Alba, Lin Dong, Rachid Chafi, Steph B. J. Menken, Merijn R. Kant

**Affiliations:** ^1^ Evolutionary and Population Biology, Institute for Biodiversity and Ecosystem Dynamics, University of Amsterdam, Amsterdam, Netherlands; ^2^ State Key Laboratory of Rice Biology & Ministry of Agriculture Key Lab of Molecular Biology of Crop Pathogens and Insects, Institute of Insect Sciences, Zhejiang University, Hangzhou, China

**Keywords:** life cycle, ontogenetic niche shift, plant defense, effector, suppression, induction, spider mite, tomato

## Abstract

When plants detect herbivores they strengthen their defenses. As a consequence, some herbivores evolved the means to suppress these defenses. Research on induction and suppression of plant defenses usually makes use of particular life stages of herbivores. Yet many herbivorous arthropods go through development cycles in which their successive stages have different characteristics and lifestyles. Here we investigated the interaction between tomato defenses and different herbivore developmental stages using two herbivorous spider mites, i.e., *Tetranychus urticae* of which the adult females induce defenses and *T. evansi* of which the adult females suppress defenses in *Solanum lycopersicum* (tomato). First, we monitored egg-to-adult developmental time on tomato wild type (WT) and the mutant *defenseless-1 (def-1*, unable to produce jasmonate-(JA)-defenses). Then we assessed expression of salivary effector genes (effector *28*, *84, SHOT2b*, and *SHOT3b*) in the consecutive spider mite life stages as well as adult males and females. Finally, we assessed the extent to which tomato plants upregulate JA- and salicylate-(SA)-defenses in response to the consecutive mite developmental stages and to the two sexes. The consecutive juvenile mite stages did not induce JA defenses and, accordingly, egg-to-adult development on WT and *def-1* did not differ for either mite species. Their eggs however appeared to suppress the SA-response. In contrast, all the consecutive feeding stages upregulated SA-defenses with the strongest induction by *T. urticae* larvae. Expression of effector genes was higher in the later developmental stages. Comparing expression in adult males and females revealed a striking pattern: while expression of effector *84* and *SHOT3b* was higher in *T. urticae* females than in males, this was the opposite for *T. evansi*. We also observed *T. urticae* females to upregulate tomato defenses, while *T. evansi* females did not. In addition, of both species also the males did not upregulate defenses. Hence, we argue that mite ontogenetic niche shifts and stage-specific composition of salivary secreted proteins probably together determine the course and efficiency of induced tomato defenses.

## Introduction

Plants possess multilayered defenses against herbivores. These defenses may be constitutively present or be induced upon attack and serve to limit damage inflicted by the herbivore (Walling, 2000). Induced defenses include morphological reinforcements and accumulation of toxins and inhibitors of herbivore food digestion (Kessler and Baldwin, 2002). In addition, plants sometimes also establish so-called indirect defenses by attracting and/or arresting foraging predators or host seeking parasitoids, e.g., *via* the production of volatile attractants or the provision of shelter or alternative food (Sabelis, 1999; Sabelis et al., 2001). These defenses are regulated mainly by two central phytohormones: (a) jasmonic acid (JA) which orchestrates the defenses against herbivores (Howe and Jander, 2008) and necrotrophic pathogens (Glazebrook, 2005) and (b) salicylic acid (SA) which primarily organizes defenses against biotrophic pathogens and phloem-feeding herbivores (Kaloshian and Walling, 2005). The actions of these two central hormones are fine-tuned by a suite of ancillary hormones and their interplay is tightly linked to the local biotic and abiotic conditions, the plant’s developmental stage and the particular tissues being attacked. SA- and JA-dependent responses were often—but not always—found to act antagonistically (Mur et al., 2006) and this was suggested to reflect an adaptive tailoring of distinct defenses against distinct attackers (Thaler et al., 2012). Feeding activities by several herbivores, e.g., aphids, whiteflies, and spider mites are known to induce both JA- and SA-dependent defense pathways (Moran and Thompson, 2001; Ament et al., 2004; Zhang et al., 2013; Cao et al., 2014). However, some herbivores can suppress the induction of plant defenses (Musser et al., 2002; Zarate et al., 2007; Kant et al., 2008; Kant et al., 2015). The generalist spider mite *Tetranychus urticae* Santpoort-2 has been shown to induce both JA- and SA-regulated defenses and produces a lower number of eggs on tomato WT plants than on JA-biosynthesis mutant *defenseless-1* (*def-1*) (Li et al., 2002; Kant et al., 2008; Alba et al., 2015; Staudacher et al., 2017). In contrast, the spider mite *T. evansi* Viçosa-1 was found to suppress the induced defenses of tomato plants (Sarmento et al., 2011b; Alba et al., 2015; Schimmel et al., 2018). However, suppression brings opportunities for non-suppressor mites to benefit from the lowered defenses when feeding on the same patch (Kant et al., 2008; Sarmento et al., 2011a; Glas et al., 2014; Alba et al., 2015; Schimmel et al., 2017a; Schimmel et al., 2017b) giving rise to complex community interactions (Blaazer et al., 2018).

Spider mites (Acari: Tetranychidae) are stylet-feeding arthropods. Unfertilized females can produce male offspring through arrhenotokous parthenogenesis, but when fertilized their offspring is a mixture of both sexes (Wrensch, 1985; Carrière, 2003). They use their stylets to pierce plant cells, predominantly parenchyma, and to inject saliva in their host. Subsequently, they ingest and digest the cell contents (Tomczyk and Kropczynska, 1985; Bensoussan et al., 2018), which leads to visible chlorotic spots on the leaf surface of the plant (Kant et al., 2004; Bensoussan et al., 2016). The two-spotted spider mite, *T. urticae*, is highly polyphagous and can be found on numerous host-plant species (Helle and Sabelis, 1985; Dermauw et al., 2012). Due to its high reproductive output (around 5–15 eggs per day, mostly depending on temperature, female age and host quality); its short generation cycle (around 14 d from egg to adult, mostly depending on temperature); its ability to rapidly adapt to novel hosts (>1,000 species recorded) and its ability to develop resistance to pesticides rapidly, this mite causes significant damage to crops worldwide (Fry, 1989; Agrawal, 2000; Van Leeuwen et al., 2010). On the contrary, *T. evansi* is more specialized and feeds predominantly on Solanaceae. It is widely present in South America and became invasive in Africa in the 1970s and, more recently, also in Europe (Ferragut et al., 2013). It is a threat to tomato cultivation as no biological control agents are available to control it (Sarmento et al., 2011b; Navajas et al., 2013).

How plants perceive spider mites and mount specific defenses is still largely unclear. First, plants may respond to the mechanical stress due to spider mite feeding and the subsequent collapse of host cells (Bensoussan et al., 2016). Mechanical injury is well known for eliciting repair and defense responses (Mithöfer et al., 2005; Duran-Flores and Heil, 2016). Second, plants may respond to spider mite egg-deposition as has been demonstrated for the eggs of dipteran (Hilker et al., 2002; Bittner et al., 2017), lepidopteran (Fatouros et al., 2015), and coleopteran (Doss et al., 2000) insects, and was shown to sometimes benefit the insect (Hilker and Fatouros, 2015; Hilker and Fatouros, 2016). Third, plants may respond to spider mite secretions such as silk (Grbic et al., 2011; Doğan et al., 2017), feces (Santamaria et al., 2015), and especially the saliva they inject into host cells during feeding, reminiscent of herbivorous insects (Howe and Jander, 2008; Maffei et al., 2012). The saliva of *T. urticae* (Jonckheere et al., 2016) and *T. evansi* (Huang et al., 2019) contains roughly 100 proteins. A family of 13 secreted salivary *T. urticae* proteins, referred to as SHOT, was shown to be exhibit strong host-dependent transcriptional plasticity (Jonckheere et al., 2018). Moreover, two additional secreted spider mite proteins, referred to as tetranins, were shown to upregulate plant defenses (Iida et al., 2019). In contrast, two salivary proteins, referred to as effector 28 and 84, were shown to suppress plant SA (Villarroel et al., 2016) and JA defenses (Schimmel et al., 2017b). How these proteins cause their effects on plants is still unknown but it has been suggested that plant receptor-like proteins may play a central role in the recognition of spider mite feeding (Zhurov et al., 2014; Santamaria et al., 2019).

The ontogenetic niche concept of Werner and Gilliam (1984) states that the use of resources of an organism depends on its developmental stage. It follows that if such resource is another organism, the ontogenetic niche shift of one may modulate the response of the other. For example, plants may respond differently to the consecutive life stages of a herbivore. The spider mite starts its life-cycle like an egg followed by four feeding stages: larva, protonymph (first nymphal stage), deutonymph (second nymphal stage), and finally the adult and these can be male or female. These stages obviously differ not only in size and morphology (Sabelis, 1985) but also in the amount of food they need and the plant tissue or cell types they are able to utilize. In addition, the stylet of juvenile spider mites may be too shallow for reaching the palisade parenchyma (the cell type mites prefer to eat) especially when residing on the abaxial (lower) leaf surface (Bandurski et al., 1953; Bensoussan et al., 2018). Another clear difference is that adult females need to eat enough to produce eggs (roughly half of their body weight per day) while males (roughly eight times smaller than females) (Mitchell, 1973) and juveniles do not.

Spider mites are small (≤0.5 mm) yet the adult females can be seen by the experienced naked eye; they are easy to distinguish from the other stages and are easier to handle than the smaller stages. In addition, the eggs laid by (young) females are considered a reliable proxy for host-plant quality; for mite population growth and for mite fitness. In standardized experiments on plant-mite interactions therefore (young) adult females are often used as representatives of the species as a whole (Li et al., 2002; Alba et al., 2015). Here we tested the robustness of this explicit assumption by monitoring the responses of the different spider mite developmental-stages to plant defenses as well as the cumulative responses of the plant to these consecutive developmental stages, similar to what will happen under natural conditions during the early stages of host colonization. We first followed the duration of the developmental stages of the two most common mite phenotypes on tomato: the first being maladapted to tomato and an inducer of tomato defenses (*T. urticae* Santpoort-2) and the second being adapted to tomato and a suppressor of tomato defenses (*T. evansi* Viçosa-1) (Alba et al., 2015) on WT tomato and on the mutant *def-1*. Subsequently, we submitted tomato plants to mite eggs and assessed the plant’s cumulative defense response, in tandem with the mite’s effector gene-expression, during the course of the mite’s development into adulthood. Finally, we compared defenses induced by young adult males with those induced by young adult females and assessed effector-gene expression in the adult sexes.

## Material And Methods

### Plants and Mites

Seeds of tomato *Solanum lycopersicum* cv. Castlemart (WT) and jasmonate acid (JA) biosynthesis mutant *defenseless-1* (*def-1*, which is in the genetic background of cv. Castlemart) were germinated and grown in the greenhouse at 25°C, L16:D8 h, 50–60% relative humidity (RH). Three days before performing experiments, plants were transferred to a climate room (25°C, L16:D8 h, 60% RH, 300 μmol m^−2^s^−1^). The two-spotted spider mite *T. urticae* Santpoort-2 (for a detailed description of this strain see: Alba et al., 2015) was maintained on detached leaves of *Phaseolus vulgaris* cv. Speedy in a climate room (25°C, L16:D8 h, 60% RH, 300 μmol m^−2^s^−1^). The red spider mite *T. evansi* Viçosa-1 (for a detailed description of this strain see: Alba et al., 2015) was maintained on detached leaves of *S. lycopersicum* cv. Castlemart placed on wet cotton wool in a climate room (25°C, L16:D8 h, 60% RH, 300 μmol m^−2^s^−1^).

### Developmental Time, Survival, and Sex Ratio of *T. urticae* and *T. evansi* on WT and *def-1* Tomato

Developmental time, survival, and mite sex ratio were determined using single mites on leaf discs. Leaf discs (15 mm in diameter) were obtained from the leaflets of 28-d-old WT and *def-1* tomato plants using a metal hole puncher. The leaf disks were placed gently (with their adaxial side up) on a wet sponge covered with wet cotton wool in a plastic tray half filled with water. Leaf disks were infested with a single egg. These had been obtained by first habituating gravid females of *T. urticae* Santpoort-2, and *T. evansi* Viçosa-1, randomly taken from the mass rearings, on intact WT and *def-1* plants for 72 h. Then single habituated females were placed on a leaf disc and allowed to produce eggs for 12 h. From each leaf disk we removed all the females and removed all eggs except one. Subsequently we monitored each of these single eggs per leaf disk for egg hatching (egg survival) and survival and development of the feeding mite stages per disc were recorded twice per day at 8- and 16-h intervals until the mites reached adulthood or died. The developmental stage was determined by observing the shed skin of the previous life stage. We recorded the sex of the adults. For each of the four treatments we monitored 100 individual mites (i.e., 100 leaf discs). After 7 d, mites were transferred to a fresh leaf disc. This experiment was repeated three times independently in time, and the data were pooled for analysis. Developmental time was analyzed per life stage comparing WT and *def-1* data for the two mite species separately using the Student’s t-test. The fraction of eggs that made it to adulthood on WT compared to *def-1* was determined after 384 h and the fraction of females relative to males among these adults on WT compared to *def-1* were analyzed separately for the two mite species after arcsine square root transformation using the Student’s t-test in IBM SPSS Statistics 25.

### Collection of Mite and Tomato Material for Gene Expression Analysis

To obtain the material for simultaneous isolation of mite RNA and plant RNA we sampled leaflets of tomato plants infested with the consecutive mite life stages (Schimmel et al., 2017a). We monitored the effect of each developmental stage on tomato defense gene expression as a cumulative effect, i.e., we included the effect(s) of the previous stage(s) by infesting plants with eggs and sampling leaflets at the end of each of the consecutive developmental stages, i.e., at the end of the egg, larval, protonymph, and deutonymph stage and at the 2-d-old adult stage. One day before starting the experiment, we took random females from the mite rearing to put on new leaflets to collect their eggs. The next day we transferred 50 eggs to the second nonterminal leaflet of the third fully expanded leaf of 28-d-old WT plants using a soft brittle paintbrush. Control plants were touched 50 times in a similar manner with a clean brush. Lanolin was put around the petiolule of the leaflets of control and infested plants to prevent mites from escaping during the course of the experiment. To determine the transition of one mite stage into the other we used a parallel “experiment” on leaf disks. We prepared 60 leaf disks (with their adaxial side up) on a wet sponge covered with wet cotton wool in a plastic tray half filled with water and placed on each disc one egg. The disks were observed twice per day and the first mite on these disks that entered the next developmental stage—as shown by their shed skin—determined the moment we sampled the intact plants. Doing so we reasoned that we would sample the intact plants at the end of each mite developmental phase under the assumption that mite development on disks and intact leaflets is similar. The disks were observed until the mites had reached adulthood. Per developmental stage we sampled the infested leaflet of five plants (five distinct biological samples), and in parallel we sampled an uninfested leaflet of five control plants (five distinct biological samples) for each stage. This experiment was repeated four times independently in time. For sampling leaves induced by one of the two adult sexes we used a different protocol based on Alba et al. (2015). Briefly, eggs were allowed to hatch on intact plants and we waited until adults were 16 d old after oviposition. We then placed 15 adult mites, either males or females, to the second nonterminal leaflet of the third fully expanded leaf of 28-d-old WT plants using a soft brittle paintbrush and sampled these after 2 d of infestation. Per adult sex we sampled the infested leaflet of five plants (five distinct biological samples), and in parallel we sampled one uninfested leaflet from five control plants (five distinct biological samples) per stage. This experiment was repeated two times independently in time. All samples were flash frozen in liquid nitrogen and stored at −80°C until we extracted mRNA.

### Expression Analysis of Mite and Tomato Genes

RNA isolation, cDNA synthesis, and assessed transcript accumulation by means of RT-qPCR were performed as described in Kant et al. (2004) and Alba et al. (2015) using the protocol of Verdonk et al. (2003). For *PI-IIc* (SGN Solyc03g020050.2), *PR-1a* (SGN Solyc09g007010.1), *Actin* (SGN Solyc03g078400.2), *RP49* (GenBank XM_015934205.2), *84* of *T. evansi* (GenBank KT182961), and *T. urticae* (GenBank XM_015936396.2) and for effector 28 of *T. urticae* (Genbank XM_025162299.1) we used the same primers as in Schimmel et al. (2017a). For *SHOT2b* of *T. urticae* (GenBank XM_015940069.1) we used the following primers: Fw GATCTTCGCCGGAAAACAAT and Rev TCATCTTCCATGAACATTAGATTGA. For *SHOT3b* of *T. urticae* (GenBank XM_015931098.1) we used the following primers: Fw TCGCCTCAACTGGAGCTT and Rev AGCAAGAGATGAACCGATTTG. For *SHOT3b* of *T. evansi* (GenBank MH979735.1) we used the following primers: Fw. GAAAATGGAGTCGCAACTGTC and Rev. ACCGAAAGTTGATAGGACACC. Quantitative PCR reactions were performed on each sample twice (two technical replicates per sample). The expression value per sample was calculated as the average of the two technical replicates. Expression was normalized using the tomato housekeeping gene *Actin* for all qPCRs because the expression of the mite housekeeping gene *RP49* varied too much during spider mite development (see *Results*). Expression was also corrected for mite survival. For the figures, the normalized transcript abundances were scaled by dividing all values including standard errors by the lowest average value (setting the latter to 1 in a data neutral manner). Data were analyzed by means of a generalized linear model, assuming gamma distribution and a log link function. The independent time points at which experiments were repeated were used as random factor in the analysis. Means of each group were compared by LSD *post hoc* test in IBM SPSS Statistics 25.

## Results

### Marginal Effects of JA-Defenses on Developmental Times of Consecutive Spider Mite Life-Stages

To assess the extent to which stage-specific developmental times of inducer and suppressor mites were affected by JA-dependent defense, we monitored the duration of the larval and the two nymphal stages of *T. urticae* and *T. evansi* males and females on leaf disks of Castlemart tomato plants (WT) and on disks of the JA-biosynthesis mutant *def-1*. The overall developmental time from egg to adult WT and *def-1* did not differ significantly for *T. urticae* Santpoort-2 (**Table 1**; *t* = 0.31, *P* = 0.76) or for *T. evansi* (**Table 1**; *t* = 0.882, *P* = 0.40) and such differences were also not seen when analyzing males and females separately (*T. urticae* Santpoort-2 females: *t* = −0.582, *P* = 0.56; males *t* = 1.157, *P* = 0.25; for *T. evansi* Viçosa-1 females: *t* = 0.562, *P* = 0.58; males: *t* = 0.686, *P* = 0.50). We did also not observe clear differences across mite species at the level of developmental stages. *T. urticae* Santpoort-2 did not exhibit significantly different developmental times for any of the stages or of the sexes on either WT or *def-1* (larva female: *t* = −1.269, *P* = 0.21; larva male: *t* = 0.577, *P* = 0.57; protonymph female: *t* = −1.179, *P* = 0.24; protonymph male: *t* = 0.811, P = 0.42; deutonymph female: *t* = -1.074, *P* = 0.29). For *T. evansi* the female protonymph stage lasted longer on WT (*t* = −2.216, *P* = 0.03). Interestingly, the developmental times of all nymphal stages of *T. evansi* males were significantly shorter on WT (**Table 1**; protonymph: *t* = 3.118, *P* = 0.003; deutonymph: *t* = 0.2873, *P* = 0.006). Also egg-to-adult survival was similar across the treatments (*F* = 1.950, *P* = 0.159). Finally, the sex ratio did not significantly differ across the treatments (**Table 1**; *F_5,12_* = 0.43, *P* = 0.819).

**Table 1:** Cumulative duration of the developmental stages, the egg-to-adult survival and the sex ratio of *Tetranychus urticae* Santpoort-2 and *T. evansi* Viçosa-1 on WT and *def*-1 tomato plants.

Treatment	Sex	Larva (hrs.)	Protonymph (hrs.)	Deutonymph (hrs.)	Adult (hrs.)	Fraction eggs reaching adulthood	Fraction adult females
*T. urticae* Santpoort-2							
*def-1*	♀+♂	147.1 ± 2.4 a	214.3 ± 2.2 a	259.5 ± 2.0 a	318.9 ± 2.7 a	0.5 ± 0.3 a	0.6 ± 0.02 a
WT	♀+♂	149.6 ± 2.6 a	216.1 ± 3.1 a	260.9 ± 2.9 a	317.5 ± 3.4 a	0.5 ± 0.2 a	0.6 ± 0.02 a
*def-1*	♀	144.9 ± 3.5 a	212.0 ± 3.3 a	258.0 ± 2.7 a	317.8 ± 3.9 a		
WT	♀	152.3 ± 4.7 a	219.2 ± 5.7 a	264.3 ± 5.2 a	321.9 ± 6.0 a		
*def-1*	♂	150.0 ± 3.1 a	217.2 ± 2.9 a	261.4 ± 2.8 a	320.2 ± 3.6 a		
WT	♂	147.6 ± 2.7 a	213.7 ± 3.2 a	258.3 ± 3.3 a	314.1 ± 3.8 a		
*T. evansi* Viçosa-1							
*def-1*	♀+♂	153.0 ± 1.9 a	213.9 ± 2.2 a	256.3 ± 2.6 a	315.5 ± 2.6 a	0.6 ± 0.2 a	0.7 ± 0.11 a
WT	♀+♂	152.4 ± 1.2 a	213.2 ± 1.5 a	251.4 ± 1.8 a	312.6 ± 2.4 a	0.7 ± 0.2 a	0.7 ± 0.08 a
*def-1*	♀	149.4 ± 2.1 a	209.0 ± 2.1 a	252.4 ± 2.8 a	317.4 ± 3.1 a		
WT	♀	152.2 ± 1.5 a	215.0 ± 1.7 b	253.0 ± 1.9 a	315.0 ± 2.8 a		
*def-1*	♂	162.5 ± 3.7 a	227.1 ± 5.1 a	266.8 ± 5.4 a	311.4 ± 4.6 a		
WT	♂	152.8 ± 1.8 a	208.8 ± 3.0 b	247.5 ± 4.0 b	306.3 ± 4.7 a		

The columns “Larva”, “Protonymph”, “Deutonymph” and “Adult” indicate the average duration in hours (hrs) it took to reach these stages from the start of the experiment. This experiment was conducted three times independently, each time starting with 100 eggs, each on a single leaf disc, per mite species per plant genotype. “Fraction eggs reaching adulthood” was calculated as the fraction of living adults after 384 h relative to the number of eggs that had been submitted to the test. “Fraction female” refers to the sex ratio expressed as the fraction of adult females. Statistics were applied to def-1 and WT data pairs in each column using Student’s t-test at α = 0.05 and data pairs marked with the same letter are not significantly different.

### Feeding Juvenile Spider Mites Induce SA-, but No JA-, Responses in Tomato

**Figure 1:**
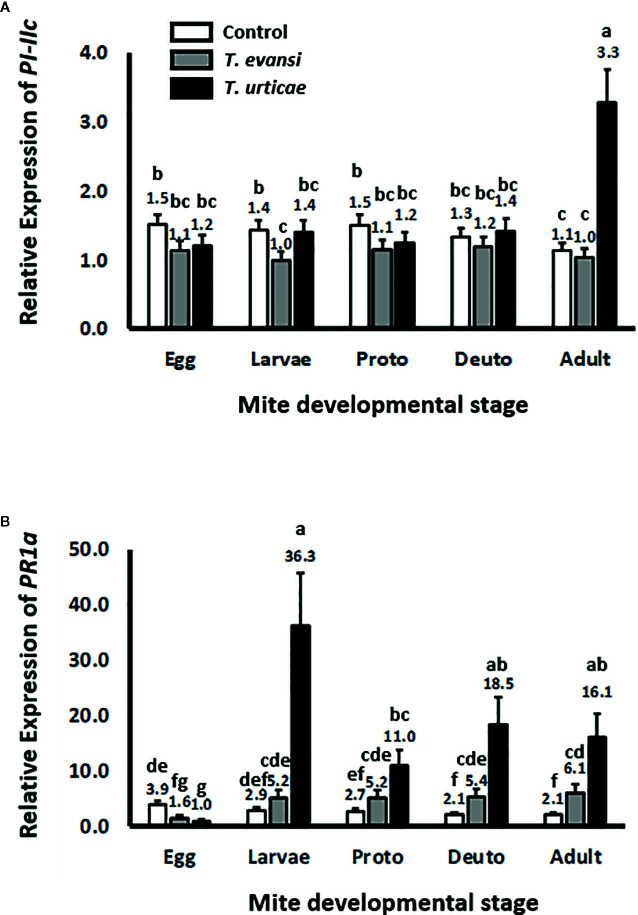
Relative expression of tomato defense marker-genes in response to the consecutive mite developmental stages (from egg to adult). Gene expression was normalized to actin. **(A)** *PI-IIc* encodes a member of the proteinase inhibitor II family and is a marker of the JA pathway. **(B)** *PR-1a* encodes a pathogenesis-related protein and is marker of the SA pathway. “Proto” stands for protonymph and “deuto” stands for deutonymph. The stages are a mixture of males and females. Sample size (n) =20 per bar. Bars with a different letter indicate a significant difference according to LSD *post hoc* test after ANOVA.

To assess whether tomato plants respond differently to different spider mite life stages we infested tomato leaflets with 50 spider mite eggs and monitored the expression of tomato genes *PI-IIc* and *PR-1a* during the course of the development of the mites from egg to adult (**Figure 1**). Overall *PI-IIc* expression was significantly affected by mite infestation (Wald χ^2^ = 54.216; *P* < 0.001). The manually deposited egg batches of either *T. urticae* Santpoort-2 or *T. evansi* Viçosa-1 did not significantly affect the expression of *PI-IIc* (**Figure 1A**). Subsequently, the larvae of *T. evansi* Viçosa-1, but not those of *T. urticae* Santpoort-2, downregulated *PI-IIc* expression. However, expression of *PI-IIc* remained near control levels during all subsequent developmental stages of both mite species until the adult stage was reached. As adults, only *T. urticae* Santpoort-2, but not *T. evansi* Viçosa-1, upregulated *PI-IIc*. In contrast to *PI-IIc*, the manually deposited eggs of *T. urticae* Santpoort-2 as well as *T. evansi* Viçosa-1 downregulated expression of *PR-1a* relative to control plants (**Figure 1B**). However, all feeding stages of both species upregulated *PR-1a* expression but *T. urticae* Santpoort-2 stronger than *T. evansi* Viçosa-1 (Wald χ^2^ = 47.292; *P* < 0.001).

#### Expression of Housekeeping Gene *RP49* Is Variable Across Spider Mite Developmental Stages

Expression of *T. evansi* Viçosa-1 or *T. urticae* Santpoort-2 genes by means of RT-qPCR is often normalized using housekeeping gene *RP49* (Morales et al., 2016; Villarroel et al., 2016; Schimmel et al., 2017a; Suzuki et al., 2017; Jonckheere et al., 2018; Yoon et al., 2018). However, Yang et al. (2015) warned that expression of *RP49* and other housekeeping genes may not be suitable for normalizing gene expression levels across developmental stages. Indeed, the levels of *RP49* expression we observed differed greatly between life stages. For *T. urticae* Santpoort-2 the average Ct (cycle threshold) of *RP49* in eggs was 30; in larvae and protonymphs 28 and in the other stages 27 (so a eight-fold difference between eggs and adults). Similarly, for *T. evansi* Viçosa-1 the average Ct of *RP49* in eggs was 29; in larvae and protonymphs 27 and in the other stages it was 25 (so a 16-fold difference between eggs and adults). Hence *RP49* was unsuitable to correct for sample-to-sample variation—i.e., variation in reverse transcription and PCR efficiency—in cDNA samples obtained from different developmental stages. However, mite RNA and tomato RNA had been collected together as total RNA (Schimmel et al., 2017a) and hence we could use tomato actin to correct for technical variation between samples. This illustrates an advantage of collecting plant and mite RNA together although it will come at the expense of mite genes with low absolute expression levels.

### Effector *84* and *SHOT3b* Genes Are Expressed Higher in Nymphs and Adults Than in Eggs and Larvae

**Figure 2:**
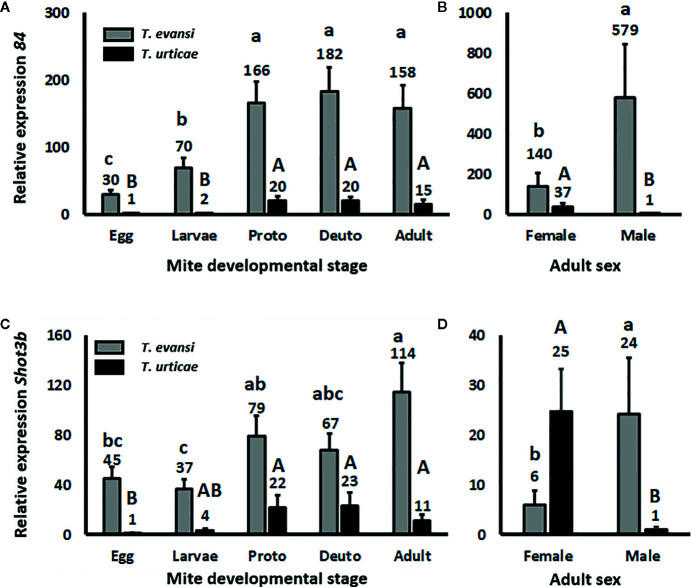
Relative expression of the mite effector gene *84* and *SHOT3b* in the consecutive spider mite developmental stages. Gene expression was normalized to actin. **(A)** Effector *84* expression in the developmental stages of *T. urticae* and *T. evansi.* **(B)** Effector *84* expression in *T. urticae* and *T. evansi* females and males. **(C)** *SHOT3b* expression in the developmental stages of *T. urticae* and *T. evansi.* **(D)** *SHOT3b* expression in *T. urticae* and *T. evansi* females and males. “Proto” stands for protonymph and “deuto” stands for deutonymph. The developmental stages in **(A, C)** are a mixture of males and females derived from 50 eggs and corrected for survival. The sample size (n) =20 per bar in **(A, C)**. **(B, D)** were conducted with 15 individuals per treatment. The sample size (n) = 10 per bar in **(B, D)**. We divided the values in **(A, B)** by the lowest average to make relative expression comparable across the two panels. The same we did for **(C, D)**. Different letters above the bars denote significant differences according to the LSD *post hoc* test (p < 0.05) after analysis by Generalized Linear Model performed per species independently.

To assess whether spider mite effector-gene expression is plastic across their life stages, we infested tomato leaflets with 50 spider mite eggs and monitored the expression of salivary effector *84* (**Figure 2A**) and *SHOT3b* (**Figure 2C**) during the course of the development of the mites from egg to adult. The expression of effector *84* per *T. evansi* Viçosa-1 individual changed during development (Wald χ^2^ = 39.872; *P* < 0.001): it increased from egg to larva and from larva to protonymph but remained stable for the later life stages (**Figure 2A**). The expression of *SHOT3b* in *T. evansi* Viçosa-1 also changed during development (Wald χ^2^ = 18.672; *P* = 0.001) yet was not significantly different between egg, larva and deutonymph (**Figure 2C**). The pattern of expression of effector *84* in *T. urticae* Santpoort-2 individuals was similar to that of *T. evansi* Viçosa-1 individuals albeit at 10–30 fold lower levels (**Figure 2A**). Also the expression pattern of *SHOT3b* in *T. urticae* Santpoort-2 individuals was similar to that of *T. evansi* Viçosa-1 but here only the expression in eggs was significantly lower than in the feeding stages and the expression was 3–10 fold lower than in *T. evansi* Viçosa-1 except for the expression in eggs that was almost 50-fold lower (**Figure 2C**). We also assessed expression of effector *SHOT2b* but the expression of this gene cannot be detected in *T. urticae* Santpoort-2 mites feeding from tomato and is not present in the genome of *T. evansi* Viçosa-1 (Jonckheere et al., 2018). Therefore, we detected expression only in the isolated females of *T. urticae* Santpoort-2 (i.e., using the same cDNA as for **Figures 2A, C**) since these had been obtained from bean. We also assessed expression of effector 28 (Villarroel et al., 2016; Schimmel et al., 2017a). Expression of effector *28* in *T. urticae* Santpoort-2 paralleled the expression of its effector *84*. It was only detected for *T. urticae* Santpoort-2 and expression was similar across the developmental stages except that the expression in protonymphs relative to eggs was significantly eight-fold higher (**Supplemental Figure S1**). Finally, we did not include *SHOT2b* in a figure because expression was only detected in *T. urticae* Santpoort-2 females but the standard error is +/− 0.39 when the average expression is set to 1.

### Effector Genes Are Expressed Higher by *T. evansi* Males Than Females but the Opposite Applies to *T. urticae*

Effector *84* was expressed four-fold higher in *T. evansi* Viçosa-1 males compared to females whereas for *T. urticae* females this gene was expressed almost 40-fold higher than in males (**Figure 2B**). This species-specific pattern was similar for *SHOT3b* since this gene was expressed almost four-fold higher in *T. evansi* Viçosa-1 males than in females whereas in *T. urticae* Santpoort-2 females expression was 25-fold higher than in males (**Figure 2D**).

### Spider Mite Males Do Not Induce Defenses

**Figure 3:**
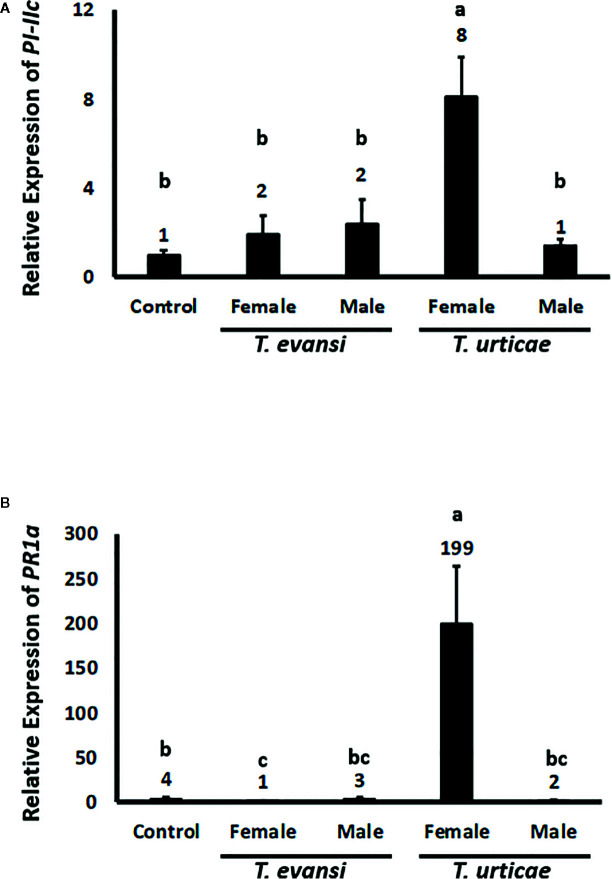
Relative expression of tomato defense marker-genes in response to 2-d old spider mite males and females. Gene expression was normalized to actin. **(A)** *PI-IIc* encodes a member of the proteinase inhibitor II family and is a marker of the JA pathway. **(B)** *PR-1a* encodes a pathogenesis related protein and is marker of the SA pathway. Sample size (n) =10 per bar. Different letters above the bars denote significant differences according to the LSD *post hoc* test (p < 0.05) after ANOVA.

To assess whether tomato plants respond differently to spider mite males or females we infested tomato plants with 15 individuals of the same sex and monitored the expression of tomato genes *PI-IIc* and *PR-1a* after 2 d (**Figure 3**). The expression of *PI-IIc* in plants infested with either *T. evansi* Viçosa-1 males or females did not exceed control levels. Also *T. urticae* Santpoort-2 males did not upregulate *PI-IIc* while females significantly upregulated its expression four-fold (**Figure 3A**). The expression of *PR-1a* was not upregulated by *T. evansi* Viçosa-1 females or *T. urticae* Santpoort-2 males. *T. evansi* Viçosa-1 females downregulated *PR-1a* expression while *T. urticae* Santpoort-2 females upregulated it 50-fold relative to the control plants (**Figure 3B**).

## Discussion

Here we demonstrated that inducible JA defenses do not significantly alter developmental time or survival of *T. urticae* Santpoort-2 and *T. evansi* Viçosa-1 males and females and do not affect the spider mite sex ratio. In addition, we showed that only *T. urticae* Santpoort-2 adult females upregulate the expression of tomato JA-marker gene *PI-IIc*, while *T. evansi* Viçosa-1 larvae downregulate the expression of this gene. Eggs of both species suppressed the expression of the tomato SA-marker gene *PR-1a* but this gene was upregulated by the cumulative action of all subsequent feeding stages, especially by *T. urticae* Santpoort-2 larvae and adult females. Expression of mite effector gene *84* was lower in eggs and larvae than in the later stages of both species and a similar pattern we observed for *SHOT3b* although differences were not always significant. In addition, in *T. evansi* Viçosa-1, expression of the effector genes was higher in males than females but for *T. urticae* Santpoort-2 this was the other way around. Furthermore, we observed that only the females of *T. urticae* Santpoort-2 induce *PI-IIc* and *PR-1a* while *T. evansi* Viçosa-1 females suppress *PR-1a* expression below housekeeping levels after 2 d of infestation. Finally, feeding by spider mite males did not alter expression of *PI-IIc* and *PR-1a*.

Since developmental time to maturity has been considered a key life-history trait for evolutionary adaptation *via* natural selection (Cole, 1954), we tested if JA-defenses affect overall developmental time of spider mites. We also analyzed this for males and females separately since males are known to develop faster than females and eat less (Sabelis, 1985; Rajakumar et al., 2005). We found that that inducible JA defenses do not significantly alter developmental time, survival of either *T. urticae* Santpoort-2 or *T. evansi* Viçosa-1 males and females and mite sex ratio. In contrast to this observation, it was shown previously that the reproductive performance of adult *T. urticae* Santpoort-2 is affected negatively by tomato JA defenses (Kant et al., 2008; Alba et al., 2015). Moreover, while the performance of tomato-adapted mites was not affected by tomato JA-defenses (Kant et al., 2008), these defenses were shown to decrease the hatching rate of their eggs (Ament et al., 2004). Finally, suppression of JA-defenses was shown to maximize fecundity of *T. evansi* Viçosa-1 (Sarmento et al., 2011b; Alba et al., 2015; Ataide et al., 2016; Schimmel et al., 2017a; Schimmel et al., 2017b). Together this indicates that JA-defenses in general have detrimental effects on adult spider mites like *T. urticae* Santpoort-2 or *T. evansi* Viçosa-1. The observation that JA defenses do not significantly alter developmental time is in line with the observation that juvenile spider mites do not induce JA-defenses. This suggests that developmental times on WT plants do not differ from those on *def-1* because juveniles do not induce this defense in WT plants. However, the juvenile feeding stages do induce cumulative SA-defenses while adult mites were found to be significantly affected by this type of defense, although the effect sizes were small (Villarroel et al., 2016). Hence, possibly spider mite developmental time may change on the tomato SA-mutant *nahG* (Glas et al., 2014). Our main conclusion is that JA-defenses seem to be much more relevant for the interaction between tomato plants and adult mites than between the plant and juveniles.

We can only speculate why JA-defenses are not induced by juveniles but we suggest it may relate to the kinds of cells/tissues the juvenile stages feed from in combination with the amount of feeding and their nutrient requirements. For example, also the juveniles of the generalist grasshopper *Schistocerca emarginata* were shown to have a much more narrow diet breadth than the adults (Sword and Dopman, 1999) while female grasshoppers were shown to often gain more weight than males (Unsicker et al., 2008) and have higher need for nitrogen for producing eggs (Chapman and Joern, 1990). Such differences may also apply to spider mites: protonymphs (3.7 μg) are three times heavier than larvae while in turn female deutonymphs are three times heavier than protonymphs (Sabelis, 1981). In addition, females are six times heavier than males (24 vs. 4 μg) and produce, depending on host quality, 5–15 eggs (1.2 μg each) per day while their estimated food conversion efficiency is around 20% (Sabelis, 1981). Clearly this indicates that females have to take up and convert much more food than males or juveniles and will be therefore probably be responsible for most of the feeding damage on the plant. Apart from nutritional needs, also mite physiology, especially stylet length, may affect the type and magnitude of the defenses juvenile mites induce. The spider mite’s feeding parts include the pedipalps and the two cheliceral stylets. The cheliceral stylets can join to form a needle-like structure used for piercing plant cells and for transferring saliva while the pedipalps contain claws for rupturing plant cell walls as well as silk glands for producing web (Ragusa and Tsolakis, 2000). The average stylet length of female *T. urticae* can vary from 103 μm (larvae) to 157 μm (adult females) (Park and Lee, 2002) and it was estimated they can reach between 70–120 μm deep into a plant leaf (Tomczyk and Kropczynska, 1985). A tomato leaflet in turn has a thickness ranging from 150 to 250 μm depending on water status and temperature (Sekhar and Sawhney, 1990; Lechowski et al., 2006; Sánchez-Rocha et al., 2008). The palisade parenchyma, the cell type mites prefer to eat, of leaflets of 170 μm thick was found to be about 20 μm under the adaxial (upper) surface but nearly 100 μm away from the abaxial (lower) leaf surface (Bandurski et al., 1953; Bensoussan et al., 2018). Since spider mites often reside on the lower leaf surface, probably to be shielded from harsh weather conditions and natural enemies, it can be difficult especially for the smaller stages to reach the palisade parenchyma. Accordingly, while chlorophyll is usually clearly visible in adults (*T. urticae* is rather transparent) it is often not in young juveniles or males. Hence larvae may feed from epidermal cells and mesophyll more than adult females do, and therefore elicit different responses, reminiscent of small mites like *Aculops lycopersici* that are also restricted to epidermal cell layers (Glas et al., 2014). Although not much is known about the abilities of different plant cell types to display JA- or SA-responses there are indications that such differences exist (Ohashi and Matsuoka, 1987; Huang et al., 1991; Uzunova and Popova, 2000) Together this indicates that ontogenetic niche shifts, e.g., characterized by a change in tissue or cell type usage by different herbivore developmental stages, may also shift the plant’s defense response.

We observed that manually deposited spider mite eggs suppressed the expression of *PR-1a* while this gene was upregulated by all subsequent feeding stages. For a variety of insect species, it was shown that their eggs can induce (Hilker and Fatouros, 2015; Hilker and Fatouros, 2016) or suppress (Bruessow et al., 2010) plant defenses (Reymond, 2013). We deposited newly produced eggs manually on the leaf surface and this may differ from natural egg deposition by female mites. At higher population densities spider mites tend to deposit most of their eggs (around 0.001 mm^3^ in size) in the web, probably to regulate egg humidity (Gerson, 1985), thereby not touching the leaf surface. When mites do deposit eggs onto the leaf surface (especially when mite densities are not so high yet) they occasionally cover these eggs with silk threads, composed of fibroin with a high serine content (Grbic et al., 2011), but there is no evidence for eggs being glued onto the leaf surface like some insects do (Voigt, 2016). Hence, the manual egg deposition we did may actually mimic natural deposition during the early stages of host plant colonization reasonably well. The egg itself has a wax layer on the outside, possibly surrounding a cement layer of oil and protein, while the embryo respires through the water resistant egg shell *via* air ducts and cone-shaped perforation organs—that are formed during embryo development—and that pierce through the shell and may conduct a lytic or plasticizing substances (Crooker, 1985). It is therefore well conceivable that substances produced during embryonic development are released on the outside of the egg; come into contact with the plant and cause physiological changes like the ones we observed. The biological significance of the *PR-1a* downregulation in response to spider mite eggs could maybe be determined using tomato SA-mutant *nahG* (Glas et al., 2014) but remains elusive at this stage. Finally, it would be interesting to assess if the mite’s endosymbiont status (Staudacher et al., 2017) of the eggs and the consecutive juvenile and adult stages change in titer and differentially affect plant defense gene expression.

We monitored the expression of four effector genes: *SHOT2b* and *SHOT3b* (Jonckheere et al., 2018) and effector 28 and 84 (Jonckheere et al., 2016; Villarroel et al., 2016). Effector *SHOT2b* is unique for *T. urticae* and only expressed in mites after eating from certain fabacean hosts like bean (*P. vulgaris*). The host-dependent regulation of *SHOT2* genes is asymmetric, i.e., it is upregulated rapidly (hours) in mites transferred to the fabacean host but down-regulated slowly (possibly only in the next generation) after transfer to a non-fabacean host (Jonckheere et al., 2018). In our experiments only the separate males and females (**Figures 2B, D**) had been obtained from bean and, accordingly, we detected *SHOT2b* expression only in these (female) mites. Hence *SHOT2b* may play a role in the *T. urticae-*tomato interaction during the early phase of the colonization (i.e., by the first generation of mites) but not likely during later generations. However, the regulation of effector *SHOT3b* is opposite to that of *SHOT2b* and is expressed higher in mites on tomato compared to mites on beans (Jonckheere et al., 2018). In our experiments on tomato, expression of *SHOT3b* was lower in eggs and larvae than in the later stages of both mite species, similar to the expression pattern of effector *28* in *T. urticae* and of *84* in both species. Unlike in earlier studies (Villarroel et al., 2016; Schimmel et al., 2017a; Schimmel et al., 2017b) we did not detect expression of effector *28* in any of the stages of *T. evansi* Viçosa-1. Possibly this was due to the fact that we collected mite and tomato RNA together as total RNA thereby diluting *T. evansi* Viçosa-1 *28* mRNA too much. The expression patterns of *SHOT3b* and effector *84* reinforce the notion that these proteins are produced and secreted primarily by the feeding stages (Jonckheere et al., 2016; Villarroel et al., 2016). Jonckheere et al. (2018) suggested the family of *SHOT3* genes to facilitate host-compatibility in a more generic manner than the *SHOT1* and *SHOT2* families. However, in contrast to *T. urticae*, expression of the *SHOT3b* and *84* genes in *T. evansi* Viçosa-1 was always higher in males than females. *T. evansi* is a gregarious species while *T. urticae* is not and possibly the *T. evansi* males play a role in creating a suitable feeding site for their kin. However, looking at the *PI-IIc* and *PR-1a* expression data also the females alone are capable of suppressing defenses (**Figure 3**) while in mixtures of males and females we observed slight yet significant *PR-1a* upregulation (**Figure 1**). Given the fact that the expression of spider mite genes associated with host defenses appeared to be rather plastic (Dermauw et al., 2012; Schimmel et al., 2017a; Jonckheere et al., 2018) it would be interesting to see how expression of effector (and detoxification) genes of *T. evansi* males is affected by the presence of related and unrelated *T. evansi* females (that both suppress defenses) as well as by the presence of defense-inducing competitors like *T. urticae* females (Schimmel et al., 2017a; Schimmel et al., 2017b). This could reveal if *T. evansi* males are capable of adjusting their magnitude of defense suppression depending on kinship with surrounding mites.

We observed that only the adult females of *T. urticae* Santpoort-2 induce expression of *PI-IIc* and *PR-1a* while adult males do not and while *T. evansi* Viçosa-1 females downregulate *PR-1a* expression below housekeeping levels after 2 d of infestation (**Figure 3**). Juveniles, on the other hand, upregulate *PR-1a* expression (**Figure 1**). These results suggest that adult males and juveniles, both being much smaller than adult females, may utilize their host plant differently than adult females. These observations also bring depth to data published previously on the timing of defense induction by adult female mites spanning a period of more than 4 d since in those samples eggs will have hatched into larvae. These larvae may account for some of the late SA responses that were observed in such time courses (e.g., Alba et al., 2015). As noted earlier, *T. evansi* males and females separately did not upregulate *PR-1a* expression (**Figure 3**) while the mixed adults (**Figure 1**) did. There are two differences between these experiments that might explain this. The first is that the total number of individuals in the life-stages experiment was about three times higher than in the male/female trial. The second is that in the life-stages experiment induction of defenses by adults was preceded by the induction of defenses by all the juvenile stages (like in nature) but in the male/female trial it was not. Both factors can have contributed to the moderate upregulation of *PR-1a* observed in the life-stages experiment. Taken together, we provided evidence that mite ontogenetic niche shifts and stage-specific composition of their saliva together may determine the course and efficiency of induced tomato defenses.

## Data Availability Statement

The original contributions presented in the study are publicly available. This data can be found here: DOI 10.6084/m9.figshare.12630299.

## Author Contributions

MK originally formulated the idea. JL, SL, and MK conceived and designed the experiments. JL, LD, JA, RC and SL performed the experiments. JL, JA, and SL analyzed the data. JL, SL, SM, and MK wrote the manuscript.

## Funding

JL was supported by the Chinese Scholarship Council (CSC). SL was supported by the Netherlands Organization for Scientific Research (STW-GAP/13550). RC and JA were supported by the Netherlands Organization for Scientific Research (STW-VIDI/13492). MK was supported under the European Union′s Horizon 2020 research and innovation program (773 902‐SuperPests and C-IPM/ALW.FACCE.6).

## Conflict of Interest

The authors declare that the research was conducted in the absence of any commercial or financial relationships that could be construed as a potential conflict of interest.
