# Supplementary material for: Juvenile Spider Mites Induce Salicylate Defenses, but Not Jasmonate Defenses, Unlike Adults
Source: Front Plant Sci. 2020 Jul 10;11:980. doi: 10.3389/fpls.2020.00980 (PMC7367147; doi:10.3389/fpls.2020.00980)
Supplement: Supplementary file 1 [file DataSheet_1.pdf]

Supplementary Material

Juvenile spider mites induce salicylate defenses, but not jasmonate defenses, unlike adults

Jie Liu, Saioa Legarrea, Juan M. Alba, Lin Dong, Rachid Chafi, Steph B.J. Menken, Merijn R. Kant*

*** Correspondence:** Corresponding Author: m.kant@uva.nl

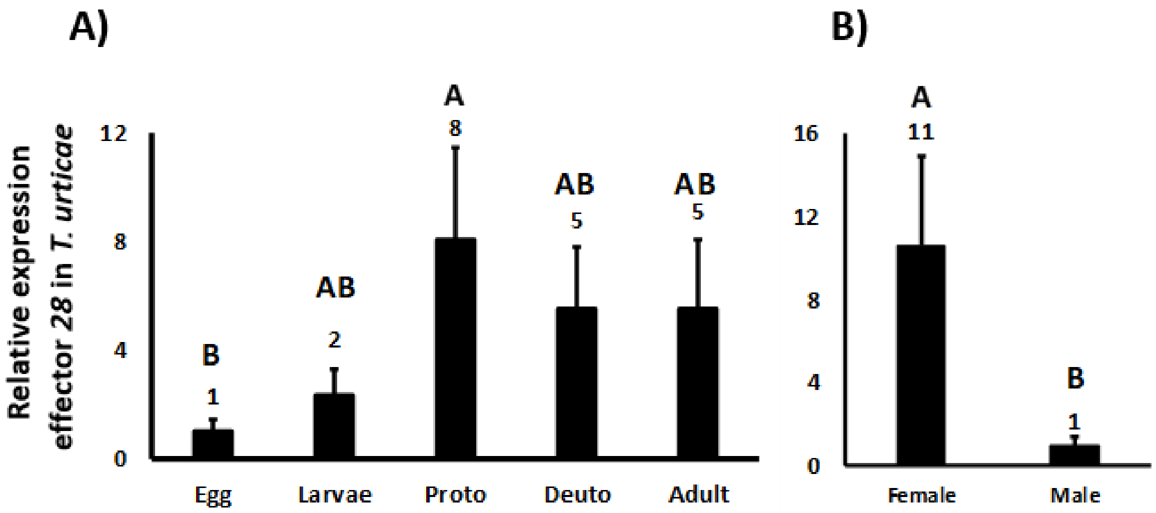

Supplemental Figure 1. Relative expression of the *T. urticae* effector gene *28* in the consecutive mite developmental stages. Gene expression was normalized to actin. **(A)** Effector *28* expression in the developmental stages of *T. urticae*. **(B)** *Effector 28* expression in *T. urticae* females and males. 'Proto' stands for protonymph and 'deuto' stands for deutonymph. The developmental stages in panel A are a mixture of males and females: they were started with 50 eggs per treatment and were corrected for survival. Panel B was conducted with 15 individuals per treatment. We divided the values in the two panels by the lowest average to make relative expression comparable across panels. Different letters above the bars denote significant differences according to the LSD post hoc test ($p<0.05$) after analysis by Generalized Linear Model performed per species independently.
